# Emerging Roles of Small Epstein-Barr Virus Derived Non-Coding RNAs in Epithelial Malignancy

**DOI:** 10.3390/ijms140917378

**Published:** 2013-08-23

**Authors:** Raymond Wai-Ming Lung, Joanna Hung-Man Tong, Ka-Fai To

**Affiliations:** 1 Department of Anatomical and Cellular Pathology, State Key Laboratory in Oncology in South China, The Chinese University of Hong Kong, Hong Kong, China; E-Mails: raymond_lung@cuhk.edu.hk (R.W.-M.L.); jtong@cuhk.edu.hk (J.H.-M.T.); 2 Institute of Digestive Diseases, Li Ka-Shing Institute of Health Sciences, The Chinese University of Hong Kong, Hong Kong, China

**Keywords:** Epstein-Barr virus (EBV), nasopharyngeal carcinoma (NPC), EBV-associated gastric carcinoma (EBVaGC), lymphoepithelioma-like carcinoma (LELC) of the lung, lymphoepithelioma-like cholangiocarcinoma, BART, miRNAs, v-snoRNA1

## Abstract

Latent Epstein-Barr virus (EBV) infection is an etiological factor in the progression of several human epithelial malignancies such as nasopharyngeal carcinoma (NPC) and a subset of gastric carcinoma. Reports have shown that EBV produces several viral oncoproteins, yet their pathological roles in carcinogenesis are not fully elucidated. Studies on the recently discovered of EBV-encoded microRNAs (ebv-miRNAs) showed that these small molecules function as post-transcriptional gene regulators and may play a role in the carcinogenesis process. In NPC and EBV positive gastric carcinoma (EBVaGC), 22 viral miRNAs which are located in the long alternative splicing EBV transcripts, named BamH1 A rightward transcripts (BARTs), are abundantly expressed. The importance of several miR-BARTs in carcinogenesis has recently been demonstrated. These novel findings enhance our understanding of the oncogenic properties of EBV and may lead to a more effective design of therapeutic regimens to combat EBV-associated malignancies. This article will review the pathological roles of miR-BARTs in modulating the expression of cancer-related genes in both host and viral genomes. The expression of other small non-coding RNAs in NPC and the expression pattern of miR-BARTs in rare EBV-associated epithelial cancers will also be discussed.

## 1. Introduction

The Epstein-Barr virus (EBV) is a ubiquitous gamma herpesvirus that was originally identified in Burkitt’s lymphoma (BL) by Tony Epstein and his colleagues in 1964 [1]. It is also the first virus known to play an essential role in the induction of human malignancies [2]. Interestingly, EBV infection is common among most population groups worldwide. However, most infected individuals are asymptomatic, and for those who show signs and symptoms of acute EBV infection tend to recover without sequelae [3]. Interestingly, the virus will not be entirely eliminated and will ultimately establish life-long latent infection within memory B cell pool, in which the viral genome circulates as episome and undergoes replication using host cellular expression machinery [4,5]. Latent EBV infection is associated with a wide range of human lymphoid and epithelial malignancies, including Hodgkin disease, Burkitt’s lymphoma (BL), nasopharyngeal carcinoma (NPC), and a subset of gastric carcinoma (GC).

While the EBV genome contains around 80 open reading frames (*ORF*s), only a few of viral proteins are selectively expressed during latent infection. Expression of these viral proteins forms four latency patterns [6]. Latency III infection is commonly observed in EBV-associated B-cell lymphoma in the setting of immunosuppression, in which all viral latent genes, including six EBV encoded nuclear antigen proteins (EBNA-1, EBNA-2, EBNA-3A, 3B, 3C and EBNA-LP) and three latent membrane proteins (LMP1, LMP2A and LMP2B), are expressed [7]. The spectrum of lymphomas in latency III includes post-transplant lymphoproliferative diseases (PTLD) and immunodeficiency associated lymphoproliferative diseases. It is likely that the immunosuppressive environment allows the expression of all latent proteins in the absence of host immune responses [8]. EBV protein expression in latency II is limited to EBNA-1 and the three LMPs. This latency type is commonly observed in NPC [9]. EBNA-1, an essential protein for virus persistence, is the only viral protein expressed in latency I. In a healthy carrier, EBV remains dormant in memory B-cells which circulate in the peripheral blood (latency 0). At this stage, expression of the EBNA-1 viral protein is found only during cell division, albeit the LMP transcript is detected at very low level in the peripheral blood [5,10]. Interestingly, two types of non protein-coding RNA, *EBV-encoded RNAs* (*EBER-1* and *EBER-2*) and long alternative splicing non-coding RNA at BamH1 A rightward transcripts (BARTs), are expressed in all forms of latency programs [11,12].

## 2. EBV Infection in Epithelial Carcinoma

### 2.1. Nasopharyngeal Carcinoma (NPC)

Nasopharyngeal carcinoma (NPC) is a distinctive type of head and neck cancer arising from the nasopharynx. This is a rare tumor in most part of the world (below 1 per 100,000 persons per year) [13]. However, the incidence and mortality rates of NPC are remarkably high in Southern China including Hong Kong (about 19.5 and 7.7 per 100,000 persons, respectively) [14]. The World Health Organization (WHO) has classified NPC into three histological categories: type I is keratinizing squamous cell carcinoma (SCC), type II is non-keratinizing carcinoma, and type III is undifferentiated carcinoma. The prevalence of individual type of NPC is dependent on geographic regions. Types I and II NPC are generally found in western population. And type III, which is consistently associated with EBV latent infection, accounts the majority of NPC in Southern China [15]. Because of the unusual prominent lymphocytic infiltration, type III NPC is also commonly described as lymphoepithelioma of nasopharynx. It has been suggested that EBV particles from the infected lymphoid cells are transferred to the nasopharynx cells via cell-cell contact [16,17]. Clonal EBV genome is consistently detected in both high-grade dysplastic lesions of the nasophargnx and invasive NPC, suggesting that EBV may contribute to the neoplastic transformation of nasopharyngeal epithelial cells and facilitate the clonal expansion of malignant cells [13,18].

Although the crucial roles of viral LMPs in NPC pathogenesis have previously been reported [19,20], their expressions in NPC are variable and generally low when compared to those in lymphoid malignancies. In NPC, viral proteins are processed and presented on the cell surface, resulting in the stimulation of host immune surveillance [21,22]. Therefore, low expression of viral proteins is critical for maintenance viral latency. In this regard, EBV may contribute to cancer development by two abundantly expressed non protein-coding viral products, *EBERs* and *BARTs*. Currently, the abundant expressions of 22 *BARTs*-derived microRNAs (miR-BARTs) in NPC have been identified; it is possible that EBV augment cancer development through these viral-encoded miRNAs [23,24].

### 2.2. EBV-Associated Gastric Carcinoma (EBVaGC)

Gastric carcinoma (GC) is another epithelial cancer reported to be associated with EBV infection. In 1990, Burke *et al.* first reported the presence of EBV DNA in a rare type of gastric carcinoma, namely the lymphoepithelioma-like carcinomas (LELCs) of stomach [25]. Subsequently, Shibata’s research team and others demonstrated that EBV is present in more than 80% of gastric LELCs [26,27]. In 1992, Shibta’s team further identified the presence of EBV in up to 16% of conventional gastric adenocarcinoma [28]. They collectively described this subtype of gastric cancer as EBV-associated gastric carcinoma (EBVaGC). Unlike NPC, EBVaGC is not an endemic disease and is present in about 10% of all gastric cancer worldwide. The incidence rates are highly similar among North America (9.9%), Europe (9.2%) and Asia (8.3%) [27,29–32]. EBVaGC represents a distinct type of GC as it has special clinicopathological characteristics such as male predominance, predisposition to the proximal stomach, lace pattern in the early GC and more frequent occurrence in gastric remnant carcinoma (GRC). EBVaGC has been usually described as latency I infection although LMP2A was weakly detected in some of the cases [29,33,34]. Previous works performed by our team also demonstrated the importance of LMP2A in gastric cancer development through genome-wide methylation analyses [35–37]. In contrast to the low expression level of viral protein, abundant miR-BARTs expression in the EBVaGC may be implicated in carcinogenesis [38,39].

### 2.3. Lymphoepithelioma-Like Carcinomas (LELCs)

LELC is histologically defined, a poorly differentiated carcinoma with dense lymphocytic infiltration. This type of cancer can be found not only in type III NPC, but also in other anatomical sites. Interestingly, EBV has been consistently detected in the LELC derived from foregut organs such as salivary gland, lung, thymus, gastric and intra-hepatic biliary epithelium (cholangiocarcinoma). They are rare cancers mainly reported in endemic regions [40–43].

LELC of the lung was first reported in 1987, and fewer than 200 cases have been reported in the literature [44]. This rare tumor occupies less than 1% of the overall lung cancer incidence in southeast China and Japan [45]. Nevertheless, EBER is consistently detected in almost all LELCs of lung in Asia. Latency program of EBV in LELC of the lung is unclear as only ~50% of the cases showed the expression of LMP1 viral oncoprotein. EBV positive carcinomas have been rarely developed in liver, some cases of these carcinomas reported as LELCs but the majority of them are EBV positive cholangiocarcinoma (CCA) [41]. In this review, we will address the expression pattern of EBV miRNAs in LELC of the lung and CCA and will discuss the possible pathogenic roles of EBV in LELC.

## 3. EBV Non Protein-Coding RNAs

### 3.1. Epstein-Barr Virus-Encoded RNAs (EBERs)

The EBERs contain two species, EBER1 and EBER2 with sizes of 166 and 172 nucleotides, respectively [46]. These two genes are separated by 161 nucleotides in the EBV genome and transcribed individually in the same direction using RNA polymerase III [46–48]. They are known to be abundantly expressed in all latent EBV infected cells with a copy number of up to 5 × 10^6^ per infected cell [46,49]. Faint EBER signals in the cytoplasm can be detected using high-resolution fluorescent *in situ* hybridization (ISH) technique with confocal laser scanning microscopy [50]. However, these transcripts are dominantly and stably confined to the nucleus, where they are associated with several ribonucleoproteins [46,51], including lupus antigen (LA) [46], ribosomal protein L22 (formerly EBER-associated protein EAR) [52–54], interferon-inducible, double-stranded RNA activated protein kinase R (PKR) [55] and retinoic acid-inducible gene I (RIG-I) [56]. As a result, EBER transcripts can be detected in all EBV positive cells by ISH and are routinely used as a probe to determine EBV infection in clinical samples. The functional role of EBERs in epithelial cancer development is not clear. Despite the demonstration of oncogenic properties of EBERs on B-cells in *in vitro* and *in vivo* experiments, EBERs can neither induce cellular transformation nor increase tumorigenicity in epithelial cell models. However, several reports indicated that EBERs can confer an apoptotic-resistant phenotype in immortalized epithelial cells and can support cell growth by stimulating insulin-like growth factor (IGF-1) secretion in EBV-positive gastric carcinoma and NPC cell lines [57–59] (for more details about EBER1 function, see [47,51]).

Early work from Steitz’s research team has shown that the predicted folding structure of EBERs is comprised of several stem-loops which are highly similar to two non-coding adenovirus-associated (VA) RNAs, namely VAI and VAII [60,61]. Moreover, EBERs can completely substitute the critical role of VA RNAs during adenovirus replication [62,63]. Intriguingly, two research groups recently demonstrated that VA RNAs can be further processed into a small functional interference RNA [64,65]. Our preliminary work also identified EBER1 derived small RNA fragments (18–25 nt) from NPC small RNA library cloning [23]. The expressions of these fragments in NPC have been verified by Northern blot analysis (Figure S1A). Nonetheless, EBER1 is unlikely to undergo typical miRNA processing due to the following reasons. Firstly, the predicted stem-loop structure on EBER1 is too small for pre-miRNA processing by Drosha/DGCR8 complex [66]. Secondly, Dicer is unable to cleave EBER1 in an *in vitro* experiment [65]. Thirdly, EBER1 is mainly confined in the nucleus and does not associate with exportin-5, a protein that facilitates nucleus export of pre-mature miRNAs [67]. We further demonstrated that EBER1 and its derived small fragments do not bind to Argonaute2 (Ago2), an integral part in the functional RNA-induced silencing complex (RISC) complex (Figure S1B).

### 3.2. Bam HI A Rightward Transcripts (BARTs)

BARTs are multi-spliced transcripts originally discovered by cDNA library analysis in C15 NPC xenograft, and their expressions have later been reported in other EBV-infected individuals [68,69]. The contribution of BARTs is dispensable in B-cell transformation since mutant recombinant EBV carrying a null BART region maintains its transforming activity in primary B lymphocyte [70]. However, it has long been speculated that BARTs have important functional roles in EBV-associated epithelial malignancies as it is abundantly expressed in NPC and EBVaGC. In the early study of BARTs, several small ORFs in the spliced cDNA transcripts were the focuses of investigation. RPMS1 and A73 were revealed to be translated into oncogenic proteins in an *in vitro* study and were shown to be negative regulators of Notch and RACK1 signaling pathways, respectively. However, expressions of these two viral proteins have not been demonstrated in natural EBV-infected samples [71,72]. Increasing interest in the functional role of BARTs, has eventually led to the discovery of 22 viral miRNAs (miR-BARTs) and a small nucleolar RNA (snoRNA) in this region [23,73–76]. The details of miR-BARTs’ function will be discussed later in this review.

### 3.3. Discovery of BART-Encoded miRNAs

MicroRNAs (miRNAs), a group of 18–24 nucleotide non protein-coding RNAs, were produced by two endogenous enzymatic digestions (Drosha/DGCR8 complex and Dicer) from the hairpin structures of long RNA transcripts. A mature miRNA recruits a group of cellular proteins, including Argonaute2 (Ago2), to form a stable complex called RNA-induced silencing complex (RISC) [77], in which miRNA acts as a guide strand to negatively regulate gene expression through imperfect complementary sequence pairing to the 3′ untranslated region (3′UTR) of the target gene [78]. One mature miRNA is expected to regulate expression of over 100 cellular genes by either repressing protein translation or inducing mRNA degradation [78]. It is estimated that about 30% of cellular protein-coding genes are regulated by miRNA [79].

**Figure 1:**
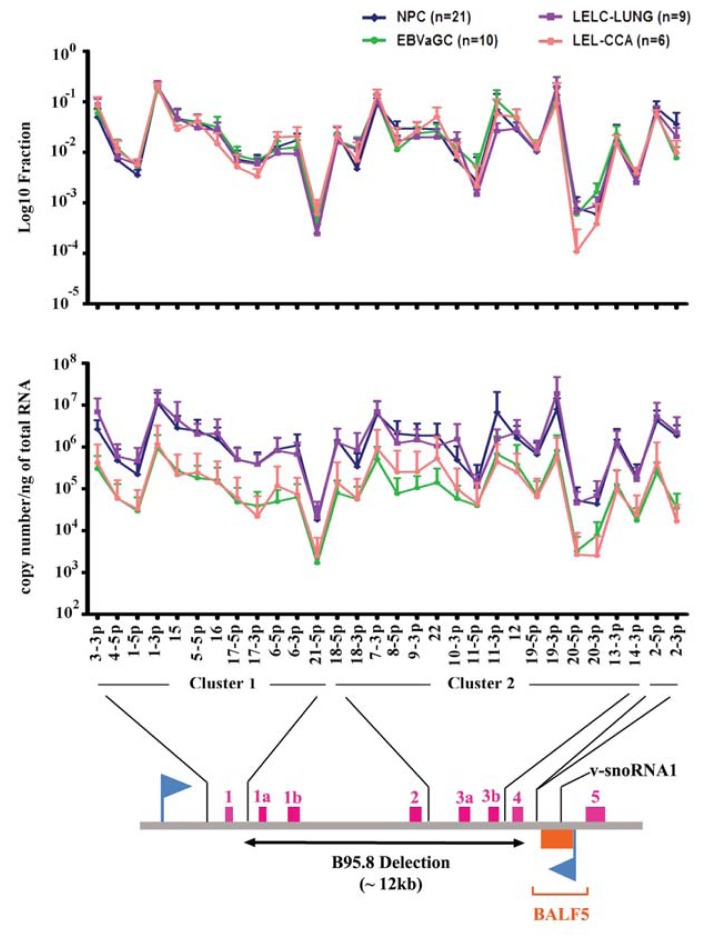
Expression of miR-BARTs in EBV positive epithelial carcinoma. The fraction of individual miRNA from all tested miR-BARTs is plotted in the upper panel. The middle panel shows the copy number of each miR-BART per nanogram of total RNA. The value represents mean + SD is plotted.

EBV was the first virus identified to encode miRNA. In 2004, Thomas Tuschl and colleagues used molecular cloning methods to identify a total of 5 miRNAs (ebv-miR-BHRF1-1, −2, −3, ebv-miR-BART1 and 2) in human B-cells infected with laboratory isolated EBV strain B95.8, which carries a 12-kb deletion in the BART region of the EBV genome [73] (Figure 1). Eventually, subsequent work on other latently infected cell lines and biopsy materials identified a total of 25 EBV encoded miRNAs [23,24,74,75,80]. Among them, three miRNAs arising from the UTR of the distinct Bcl2 homolog gene *BHRF1* (ebv-miR-BHRF1-1, −2 and −3) are predicted to be expressed in the EBV latency III program. The rest of the remaining 22 miRNAs (miR-BART1 to miR-BART22), which are located mainly in two clusters within the non-coding BART region, are generally highly expressed in most of the EBV infected epithelial cells (latency I and II) [20,39].

### 3.4. Discovery of Viral-Encoded snoRNA

Nucleoli contain a large population of conserved small stable RNAs known as small nucleolar RNA (snoRNA). They can mediate nucleotides modification of ribosomal RNAs and facilitate their production by forming a functional snoRNA:protein complex (snoRNPs) with a group of snoRNA core proteins [81]. Based on deep sequencing analysis of cDNA clones from B lymphocytes, Hutzinger *et al.* identified a snoRNA gene within the EBV genome. The viral snoRNA, mapped ~100bp downstream to the miR-BART2 transcripts, was 65 nucleotides (nt) in length and designated as v-snoRNA1. The authors also confirmed the expression of v-snoRNA1 in a panel of EBV positive BL and LCL cell lines. Given that v-snoRNA1 could associate with three canonical snoRNA proteins (fibrilarin, Nop56 and Nop58), it follows that v-snoRNA1 could be assembled into functional snoRNPs. On the other hand, a 24 nt RNA fragment from the v-snoRNA1 3′ terminus (v-snoRNA^24pp^) had been identified in cDNA library screening, but its expression was only detected during lytic induction on EBV infected 293 cells [76]. In view of the fact that some snoRNA species were reported as precursors for miRNA biogenesis [82,83], v-snoRNA1 might also be further processed into miRNA-like molecule, v-snoRNA^24pp^. However, the experimental evidence to prove this hypothesis is not yet provided.

The miRNAs possess properties to modify the intracellular environment, a characteristic that is particularly useful and convenient for viral infection. Viral miRNAs were processed from short RNA transcripts (~200 nt) by using cellular transcriptional machinery. Thus, a number of miRNA precursors can be tightly packed into the relatively small viral genome. In addition, miRNAs are non-protein coding molecules that are presumably immunogenically inert, yet they can modulate several target genes that can enhance infected cell survival.

## 4. Expression of Viral miRNAs in Epithelial Cancers

### 4.1. Ebv-miR-BHRF1s

All three ebv-miR-BHRF1s are suggested to be derived from two latency III specific Cp/Wp-initiated transcripts, the primary EBNA transcript and the latent BHRF1 transcript. For this reason, BHRF1 miRNAs are mainly expressed in this specific latency program [38,73,74,84–86]. However, several research teams using QRT-PCR technique indicated the presence of BHRF1 miRNAs in latency type II EBV positive epithelial cancer cell lines [87,88]. The inconsistent observation may be the result of active EBV replication in a small portion of infected cells. During EBV replication, miR-BHRF1-2 and −3 may be generated from the lytic form of the BHRF1 transcripts that were produced from the alternative lytic promoter BHRF1p [86]. As a gold standard, Northern blot analysis is used for the detection of miRNA expression. Surprisingly, we could detect the expression of the lytic form of the BHRF1 transcript in NPC samples including cell lines, xenografts and half of the actual patient samples. However, no expression of BHRF1 miRNAs was detected in any of the tested samples (Figure S2A). In contrast to previous studies that reported that induction of lytic EBV replication could facilitate miR-BHRF1s expression in B-cells [74,84], induction of EBV lytic cycle in the native EBV positive NPC cell lines C666-1 did not activate miR-BHRF1 expression at detectable levels (Figure S2B). This finding aligned with the previous report that BHRF1 transcription does not correlate with the expression of mature miR-BHRF1s [86]. Failure of miR-BHRF1 biogenesis may be the result of the absence of their target transcript in NPCs, leading to the sharp degradation of the miRNAs [89]. In addition, while the level of miR-BHRF1s was negligible, miR-BARTs were detected from the biopsies of EBV positive lymphoepithelioma-like cholangiocarcinoma and LELC of the lung (Figure S2C). This is in line with the previous data that BHRF1-derived miRNAs are not processed in epithelial cancers when EBV infection is mainly in latency I/II programs [24,38,80,85].

### 4.2. Ebv-miR-BARTs

Viral BARTs are initiated from two constitutively active promoters P1 and P2 [86,90]. And miR-BARTs are the only known functional molecules produced from these transcripts. By using RT-PCR and small RNA library sequencing methods, expression profiles of EBV miRNAs in cancer have been extensively studied, albeit most of the studies focused on NPC [24,39,85–88,91,92]. It is obvious in NPC that miR-BARTs are abundantly expressed and constitute up to 23% of the total miRNAs [24]. However the expression of individual miR-BART in the cells is considerably different. The comprehensive miRNA profiling study by Qiu *et al.* revealed that several miR-BARTs that were not detected in normal infected cells (germinal center B cells: latency II and memory B cells: latency 0/I) were upregulated in various EBV-associated malignancies such as BL, EBVaGC, Hodgkin Disease and NPC. These critical findings designated the importance of miR-BARTs in the development of EBV-associated cancer [87].

Qiu *et al.* further concluded that the expression patterns of EBV miRNAs from two epithelial cancers (5 NPCs and 6 EBVaGCs) were extremely similar [87]. To gain insight into the expression pattern in other epithelial cancers, our team employed home-designed QRT-PCR assays to profile the expression of EBV miRNAs in two common and two rare EBV-associated epithelial malignancies in our local primary biopsies, including NPC (*n* = 23), EBVaGC (*n* = 10), LELC of the lung (*n* = 9) and lymphoepithelioma-like CCA (*n* = 6). The presence of EBV in the samples was confirmed by EBER *in situ* hybridization (ISH) on the paraffin-embedded tissue section. Since the tumor contents and transcription activities among individual samples might be different, we measured both the absolute miRNA copy number per nanogram of total RNA and the fraction of viral miRNAs. To obtain an absolute quantification of each miRNA in the sample, an in parallel experiment using synthetic DNA-RNA chimeric oligonucleotides of each mature miRNA sequence as a template for a standard curve was included (Table S1). On the whole, all miR-BARTs were favorably expressed in all the tested EBV associated malignancies except for miR-BART20 and miR-BART21, which showed low expression levels of 10^3^ copies per nanogram of total RNA. The low expression levels of these two miRNAs aligned with our previous findings in a small-scale cloning analysis on NPC samples [23]. Whereas the average copy numbers of BART miRNAs expressed in NPC and LELC of the lung was around 5–10 fold higher than EBVaGC and CCA, the miR-BARTs fraction profile of four tumor types were vastly similar (Figure 1).

## 5. Recent Research Development of Viral Small Nucleolar RNA (v-snoRNA1)

### 5.1. Expression of v-snoRNA1 in NPC

**Figure 2:**
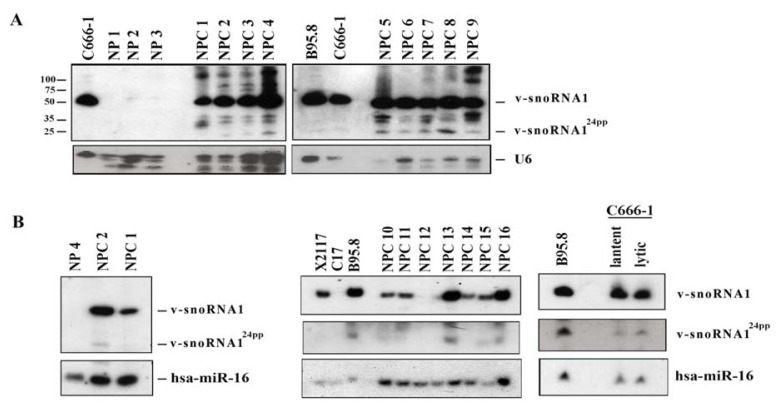
Expression of v-snoRNA1 in NPC: (**A**) Expression of v-snoRNA1 and v-snoRNA1^24pp^ on NPC biopsies (NPC 1 to NPC 9) were analyzed by Northern blot analysis. Biopsies from noncancerous nasopharyngeal tissues (NP 1 to NP 3), NPC cell lines (C666-1) and B95.8 cells were included as controls. The membranes shown in Figure 2A were used previously in our publication [93]; (**B**) To confirm the result on Figure 2A, Northern blot analysis for samples from NPC 1, NPC 2 (*left panel*) and another 7 new NPC biopsies (*middle panel*) were performed on the newly prepared membranes. The effect of EBV replication on v-snoRNA1 expression in C666-1 cells is shown in the *right panel*.

Apart from the production of miRNAs, abundant BARTs expression in epithelial cancer may have another important role by generating v-snoRNA1 and v-snoRNA1^24pp^. However, v-snoRNA1 expression is so far only described in EBV infected B-cells. In our recent study, we aimed to characterize the expression of v-snoRNA1 in NPC. We noticed in Northern blot analysis that v-snoRNA1 was highly expressed in almost all NPC samples including cell lines, xenografts and biopsies. Furthermore, v-snoRNA1^24pp^ was detected in a portion of the NPC samples (Figure 2A,B). Previous work by Hutzinger *et al.* demonstrated that inducing EBV lytic replication could upregulate both v-snoRNA1 and v-snoRNA1^24pp^ production in wild type EBV infected 293 cells [76]. In contrast, we demonstrated that in native EBV infected C666-1 cells, induction of the lytic cycle could only slightly up-regulate v-snoRNA1^24pp^ expression and had no observable effect on v-snoRNA1 expression (Figure 2B).

### 5.2. Nucleotide Polymorphism Is Important for v-snoRNA1 Production

**Figure 3:**
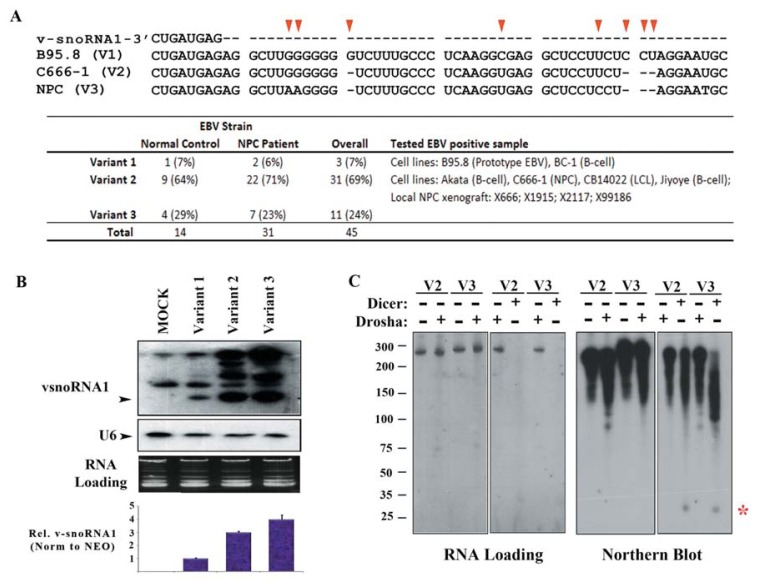
Nucleotide polymorphism of vsoRNA1 flanking sequence: (**A**) The three nucleotide polymorphism patterns (V1–V3) positioned distal from the 3′ end of v-snoRNA1 were identified. The sequence shown is corresponded to 152925:152985 (AJ507799.2); (**B**) Expression level of v-snoRNA1 was analyzed by Northern blot using RNA from 293FT cells transiently transfected with expression vectors containing different v-snoRNA1 variants as indicated (AJ507799.2, 152761:153032); (**C**) *In vitro* Dicer and Drosha/DGCR8 processing on the long v-snoRNA1 containing fragment. The digestion products were visualized on 10% PAGE (*left panel*) and analyzed by Northern blot with v-snoRNA24pp complementary oligonucleotide probe (*right panel*). The signal of v-snoRNA24pp is showed as “*”. The representative result is shown.

We next compared the v-snoRNA1 sequence in our available cell lines and NPC xenografts to the B95-8-EBVstrain by direct sequencing. Our finding was consistent with the previous observation by Hutzinger *et al.* that v-snoRNA1s from different EBV strains are highly conserved. However, several nucleotide polymorphisms at a position distal from the 3′ end of v-snoRNA1 were observed. BC-1-EBV and B95-8-EBV strains had the same sequence polymorphism (variant 1), and all the other tested samples carried another sequence pattern as the C666-1-EBV strain (variant 2) (Figure 3A). To further analyze the variation pattern of EBV strains from 14 normal controls and 31 NPC patients in our population, we showed that the variant distribution of EBV strain was highly similar between NPC patients and normal controls, in which variant 2 accounted for ~70% in our population. Another variant (variant 3) with a three-nucleotide polymorphism to variant 2 accounted for 24% in our locality (Figure 3A). EBV nucleotide variation positioned distal from the hairpin structure was previously reported to affect miR-BART processing [23].

To investigate whether the common variant would affect v-snoRNA1 production, we cloned different variants of v-snoRNA1 containing fragment (~270 nt) into the pcDNA 3.1 (+) expression vector and transiently expressed them into 293 cells. Subsequent Northern blot analysis on the RNA extracts revealed that both fragments from variants 2 and 3 displayed higher expression levels of v-snoRNA1 than the prototype EBV variant 1 (Figure 3B). Surprisingly, the predicted proximal RNA hairpin structures among these three variants are extremely similar to each other (Figure S3). In this sense, the Drosha/DGCR8 complex, which chopped off the hairpin structure (premature miRNA) from the long RNA transcripts, might not be responsible for viral v-snoRNA1 biogenesis. We further substantiated this hypothesis by performing additional *in vitro* enzymatic digestion on T7 transcribed RNA fragments from expression vectors that disabled Drosha/DGCR8 complex’s activity to generate v-snoRNA1 [23,94] (Figure 3C). Thus, the v-snoRNA1 or v-snoRNA^24pp^ biogenesis pathway might be dependent on other microprocessor complexes such as the Integrator complex, which was previously proved to take part in the viral pre-miRNA processing in primate herpesvirus [95]. On the other hand, we demonstrated that Dicer activity alone was sufficient to process the *in vitro* transcribed long RNA fragment into v-snoRNA1^24pp^. Hence, v-snoRNA^24pp^ may be directly produced from v-snoRNA1 or it longer precursor with the help of Dicer activity (Figure 3C).

## 6. Functional Roles of miR-BARTs in Cancer Development

### 6.1. Methods for miRNA Targets Identification

Viral miRNA is expected to contribute to cancer development by silencing both the viral and cellular target transcripts in the post-transcriptional level. Thus, identification and validation of the downstream targets of miR-BARTs are critical to understand how EBV is involved in tumorigenesis. It is generally accepted that a perfect match between the miRNA seed sequence (nucleotides 2–8) and the target 3′UTRs is important in the interaction. However, the exact parameter to determine miRNA target recognition is not fully characterized. As a result, a huge amount of false positive targets are predicted by using *in silico* miRNA target prediction method. To avoid considering the elusive universal rule for target site binding, high throughput sequencing of RNAs isolated by cross-linking and immunoprecipitaion (HITS-CLIP) [96] and photoactivatable ribonucleoside enhanced CLIP (PAR-CLIP) [97,98] methods have recently been employed to identify the endogenous miR-BARTs associated targets [99–101]. These biochemical methods have been reported to identify a hundred of miR-BART cellular targets, indicating the heavy involvement of EBV in regulating the cellular transcriptome. Remarkably, most of the known targets of miR-BARTs are mainly responsible for the control of viral latency, host cell immunity and apoptosis [100,101].

### 6.2. Regulation of Viral Gene Expression

Although target identification is the most challenging part in miRNA study, the targets of some viral miRNAs are easy to identify because they are transcribed as an antisense sequence of the viral genes. The first EBV miRNA identified to target viral product was miR-BART2-5p, which was transcribed in the opposite direction to the 3′UTRs of the viral DNA polymerase gene, *BALF5* [73]. With the perfect complementarity between miR-BART2-5p and BALF5 transcript, miR-BART2-5p was finally proved to down-regulate BALF5 expression via siRNA cleavage at the binding site [102]. Similarly, vsnoRNA1^24pp^, positioned distal from miR-BART2 and fully complementary to the BALF5 3′UTR, was indirectly proved to down-regulate BALF5 expression by cleaving the transcript [76]. Being a critical protein for DNA replication, down-regulation of BALF5 may facilitate EBV to enter latency.

Apart from the direct cleavage mechanism, EBV-miRNAs also regulate viral gene production by associating with their 3′UTRs. It had been revealed by our previous work in NPC that expression of LMP1 and LMP2A was downregulated by three BART cluster 1 miRNAs (miR-BART1-5p, 16 and 17-5p) and BART22, respectively [23,103]. Although both LMP1 and LMP2A can promote cell proliferation, migration and invasion, high expression of these two proteins in the infected cells may have destructive effects. LMP2A is the most immunogenic viral protein in latency I and II infection, and circulating T-cells specific for LMP2A derived epitopes are frequently detected in healthy individuals [104,105]. Limiting LMP2A expression by miR-BART22 would have potential advantages for the cells to escape host immune surveillance. On the other hand, high LMP1 expression can suppress cell growth and guide the cells to commit apoptosis in response to several stimuli like TNF treatment and serum depletion [106,107]. Thus, LMP1 expression in cancer should be tightly regulated in order to maintain the balance between its growth promoting and suppressing effects [103]. Fascinatingly, more miR-BARTs have been identified to regulate LMP1 expression in lymphoma cell lines. Ranakrishnan *et al.* showed that miR-BART9 from the NK/T-cell lymphoma cell line SNK6 could increase viral LMP1 expression. This might be the key mechanism of EBV to promote cell growth [108]. However, the mechanism behind this regulation was not clearly demonstrated. Using Ago HITS-CLIP direct biochemical assay in EBV-transformed B cells (Jiyoye cells), Riley *et al.* further recognized that both miR-BART5 and miR-BART19-5p could down-regulate LMP1 expression [101]. While miR-BART5 and hsa-miR-18-5p share the same seed sequence, hsa-miR-18-5p is unable to target LMP1 [101]. This suggested that additional factors, in addition to the perfect Watson-Crick complementary between target 3′UTR and miRNA seed sequence, should also be considered for miRNA suppressive activity [23]. Riley *et al.* further uncovered that miR-BART10-3p could work together with miR-142-3p and miR-17-5p to co-repress viral anti-apoptotic gene *BHRF1* in latency III EBV infection cells. The means to target *BHRF1* was unknown, but blocking these miRNAs individually in Jiyoye cells definitely increased the cellular apoptotic rate [101]. Table 1 lists the confirmed viral targets of miR-BARTs.

### 6.3. Regulation of Cellular Gene Expression

As a successful pathogen, EBV can develop life-long latency in the host with subsequent reactivation. For this reason, EBV needs to protect itself by tightly controlling viral gene expression and regulating host signaling cascades. It is not surprising to learn that miR-BARTs are the key regulators for host gene expression because only a few of viral proteins are expressed in carcinomas.

Until now, multiple lines of evidence have suggested that miR-BARTs target cellular genes mainly for preventing apoptosis and escaping the host immune system. The p53-up-regulated modulator of apoptosis (PUMA) is the first recognized cellular target of miR-BARTs and was targeted by miR-BART5 via the interaction on its 3′UTR [93]. The PUMA is one of the six members of the BH3-only group in the Bcl2 family. The BH3-only proteins, the essential initiators of apoptosis, are responsible to control the release of cytochrome C from the mitochondrial inter-membrane [109]. Although PUMA is the immediate downstream target of the well-known tumor suppressor gene *p53*, it functions as a key pro-apoptotic protein under the influence of the p53 tumor suppressor and other apoptotic stimuli. With apoptotic stimulation, PUMA is believed to interact with Bcl-2 family proteins and helps to mediate mitochondrial dysfunction, followed by the activation of the caspase cascade and eventually induces apoptosis. The interaction of miR-BART5 and PUMA on cell apoptotic effects was clearly demonstrated in EBV-positive NPC cell lines, in which depletion of miR-BART5 or ectopic expression of PUMA could make the cells more susceptible to apoptosis-inducing drugs [93].

Another BH3-only group protein, Bcl-2 interacting mediator of cell death (Bim), was also shown to be the target of the multiple miRNAs encoded in the BART cluster 1 [110]. Interestingly, Bim’s 3′UTR is not responsive to any of the individual miR-BARTs in stable transfectants. This observation indicates the importance of the co-operation of miR-BARTs in cluster 1 for Bim expression. In another study on EBV positive NK/T cell lymphoma, Lin *et al.* demonstrated by transient transfection and Western blot analysis that miR-BART20-5p could downregulate the T-box transcription factor TBX21 expression via the binding site located on the 3′UTR of the target mRNA [111]. They further revealed that inhibition of the TBX21 activity indirectly suppressed p53 function in NK/T lymphoma cell lines. This finding underlined the significance of miR-BART20-5p on preventing p53 dependent apoptosis. However, this miRNA is generally weakly expressed in epithelial cancers (Figure 1).

By using direct co-immunoprecipitated miRNA/mRNA containing RISC method, several studies identified a number of putative anti-apoptotic targets regulated by miR-BARTs. TOMM22 was identified and validated as a potential target of miR-BART16 [112]. This protein is a part of the mitochondrial membrane receptor involved in the association of the pro-apoptotic protein, Bcl-2-associated X (Bax). Treatment with antibodies against TOMM22 or siRNA knockdown has been shown to inhibit the association of Bax protein on mitochondria, thus preventing Bax-dependent apoptosis [113]. In Burkitt’s lymphoma, BART miRNAs were found to exert anti-apoptotic effects by repressing CASP3 expression, in which the co-suppressive effects of miR-BART1-3p and −16 on CASP3 expression was confirmed by reporter assay [114]. Riley *et al.* also reported in HITS-CLIP result that both viral miR-BART13-3p and host miR-17 could inhibit Caprin-2 translation [101]. Despite Caprin-2’s ability to promote activation of the oncogenic Wnt signaling pathway [115], over-expression of this protein would drastically induce apoptosis [116].

Surprisingly, not all miR-BARTs promote cell growth or inhibit apoptosis. Choi *et al.* demonstrated the tumor suppressive effect of miR-BART15 in gastric cancer [117]. The BRUCE protein is a member of the inhibitor of apoptosis (IAP) family that protects cells from apoptosis by ubiquitin-dependent degradation of caspase protein [118,119]. It was indicated in transient transfection assay that miR-BART15 could suppress cell growth and induced early apoptosis in AGS gastric carcinoma cell in part through targeting the *BRUCE* gene. By carrying a similar “seed sequence” with miR-223, miR-BART15 was also reported to repress NLRP3 expression via the miR-223 putative binding sites on the 3′UTR of NLRP3 [120]. NLRP3 is a NOD-like receptor protein that forms a multi-protein complex called the inflammasome. In response to pathogen invasion, NLRP3-inflammasome inside the cells can be activated to trigger the secretion of interleukin-1β (IL-1β), finally resulting in the stimulation of host inflammatory response. Thus, the presence of miR-BART15 may protect the infected cells by functioning as a NLRP3 blocker. Moreover, miR-223 has been proved as a tumor suppressive miRNA in other epithelial cancers. For example, it can suppress oncogenic protein (stathmin1) expression in liver and gastric carcinomas [121,122]. Yet, the effect of miR-BART15 on the same oncogenic proteins has not been evaluated. Further studies may be needed to uncover the complexity of the miRNA network in carcinogenesis.

Recently, several miR-BARTs in infected cells were selectively secreted as an exosome and transported to the adjacent cells to exert their suppressive function [120,123,124]. In addition, the exosomal miR-BART15 from EBV-infected B cells was found to be sufficient for the inhibition activity of the NLRP3 inflammasome in the PMA-differentiated macrophage cell line THP-1 [120]. In this sense, miR-BART15 might indirectly contribute to cancer development by exercising its “anti-cancer” activities in the adjacent immune cells [117].

Besides BART15, two more miR-BARTs were also reported to augment cancer progression by evading host immunity system. For example, EBV miR-BART2-5p has been shown to regulate the stress-induced *MICB* gene [125]. Since MICB is a key cell surface ligand for natural killer (NK) cells recognition, down-regulation of MICB expression may allow them to escape direct killing by NK cells. The innate immunity related gene *importin 7* (*IPO7*) was a putative target recognized by miR-BART1-3p and BART3 [112,114,126]. The *importin 7* gene encodes a critical receptor protein responsible for importing transcription factors into the nucleus [127]. It is believed that IPO7 is involved in the innate immunity system because knocking down of this gene in macrophage reduces pro-inflammatory cytokine IL-6 secretion [112]. The function of IPO7 in epithelial cells is largely unknown. Therefore, miR-BART3 may mainly exert its function in neighboring immune related cells via exosome.

Aside from genes implicated in apoptosis and immune evasion, a few studies independently reported the function of miR-BARTs on regulating tumor suppressor genes and the gene responsible for latency maintenance. Although the direct interaction between miRNAs and the corresponding target sites were not assessed, Wong *et al.* identified in a transient transfection assay that miR-BARTs could extensively down-regulate the expression of several well-known Wnt inhibitory molecules, such as *WIF1* (miR-BART19-3p), *APC* (miR-BART19-3p, 7 and 17-5p) and *NLK* (miR-BART19-3p, 14 and 18-5p) [92]. On the other hand, miR-BART3* has been showed to down-regulate the expression of tumor suppressive gene *DICE1* in NPC cells [128]. Since inactivation of *DICE1* occurs frequently in non-EBV associated solid tumors by either allelic deletion or CpG promoter hypermethylation [129–131], this finding has highlighted the importance of miR-BART3 to downregulate DICE1 activity in EBV related carcinogenesis.

It has been shown in primary B cells that EBV infection could globally suppress the expression of cellular miRNAs, indicating the presence of strong miRNA repressors within the EBV genome [132]. The miR-BART6-5p may be one of such EBV-derived repressor since it can inhibit the expression of human Dicer, a critical enzyme for miRNA biogenesis, via four binding sites within the 3′UTR [133]. In addition, knocking down miR-BART6-5p activity in EBV positive cell lines with latency I or II program can enhance Dicer expression and indirectly re-activate the expression of both lytic and highly immunogenic viral proteins such as Zta, Rta and EBNA2 [133]. These findings suggested that the key function of miR-BART6-5p might assist infected cells to maintain latency.

## 7. Conclusions and Future Perspectives

In NPC and EBVaGC, the clonal EBV genome is consistently detected in all invasive carcinoma and high-grade dysplastic lesions. This observation underscores the importance of viral infection in the development of epithelial cancers. In this context, EBV should have the ability to amend several cellular signaling pathways for both host cell survival and to facilitate tumor progression. In contrast to the low viral protein levels, functional BART miRNAs are generally highly expressed in a number of epithelial cancers (Figure 1). Although the regulatory mechanism by which miRNAs affect tumor progression is not fully understood, some viral miRNAs have already been recognized to regulate expression of several key cancer-related proteins, including those responsible for facilitating latency maintenance, immune suppression and tumor promotion (Tables 1 and 2). For this reason, suppressing oncogenic viral miRNAs (viral onco-miR) activity in the cancer cell may provide a therapeutic benefit for patients.

After the discovery of the first human miRNA 10 year ago, only the antagomir against miR-122 (SPC3649, Miravirsen) can enter into Phase II clinical trials for Hepatitis C treatment [134,135]. However, this therapeutic strategy is being continuously developed and improved, especially on the issues of delivery system and inhibitory efficiency of the therapeutic particles [136,137]. Currently, the schemes for targeting miR-BARTs are reviewed in great detail by Lo AK *et al.* [138]. In theory, EBV miRNAs are exogenous genes solely expressed in cancer cells, and display low sequence homology to known human miRNAs [139]. Advantage would be given to the development of miR-BART targeting therapeutic approach because of the tissue specific delivery and the potential off-target effect of the designed bullet. However, selecting suitable viral onco-miR candidates for targeting therapy is challenging. In this review, we revealed that the miR-BARTs expression patterns of four common EBV-associated epithelial cancers are highly similar (Figure 1). This observation has suggested that most EBV-positive epithelial cancers employ similar regulatory mechanisms for controlling ebv-miRNA expression, thus a single formula of miRNA inhibitor may be possible for the treatment of those malignancies. Nevertheless, our understanding of viral miRNA function is still far from complete. Despite that several important cellular viral miRNA targets have been identified, their interactions were solely substantiated in cell culture experiments in which non-physiological levels of miRNAs were used. In addition, a single miRNA molecule might be possible to regulate numerous signaling cascades in response to different cellular environments. Therefore, all the identified miRNA targets should be tested in *in vivo* experiments. We believe that a comprehensive understanding of miR-BART function and expression pattern in epithelial cancers is crucial in designing effective therapeutic regimens to combat EBV-associated malignancies.

The involvement of EBV miRNAs on cellular interactions is even more complicated. Currently, certain EBV miRNAs were shown to be actively secreted from infected cells and transported to neighboring cells through a group of small endosomal-derived vesicles called “exosomes” [124,140–142]. It had been demonstrated in *in vitro* and *in vivo* experiments that secretory exosomal ebv-miRNAs can be internalized and exhibit biological functions in the surrounding uninfected recipient cells such as primary monocyte-derived dendritic cells (MoDC) [123,124,143,144]. Thus, EBV seems to utilize the exosome as a vehicle to extend their miRNA function on the neighboring cells for immune evasion. Being protected from enzymatic degradation by membrane bound microvesicles, exosomal miRNAs are efficiently recovered from biological fluids such as plasma, pleural fluid or sputum. This characteristic has lead scientists to consider exosomal viral miRNA as a non-invasive biomarker. However, the study of exosomal miRNAs is only at the initial stage, and the underlying mechanism to regulate its secretion remains largely unclear. For example, circulating miR-BART17 was consistently detected in plasma and serum samples from NPC patients [92,141,145], yet, the reason why the same miRNA was not enriched in the exosomal fraction of the same patients was unknown [145]. In addition, the exosomal miRNA content in hepatocytes has been reported to vary between different pathological and physiological conditions [146]. Whether these conditions may affect the composition of exosomal viral miRNAs in EBV positive cancer patients is not characterized. To this end, the present information is not sufficient to support the use of exosomal content as a biomarker. Nonetheless, Arroyo *et al.* found that the majority of circulating plasma miRNAs was protected by Ago2 complexes [147]; it would be interesting to know if the HITS-CLIP method can be used for biomarker identification.

Despite the uncertainty of the nature of exosomal miRNAs, some EBV miRNAs exhibit a higher expression in the blood from NPC patients compared to non-NPC controls [92,145,148]. The preliminary work from Courzones and colleagues further revealed that the plasma concentration of miR-BART17 in NPC patients is partly dependent on the tumor mass [145]. Surprisingly, there seemed to be no correlation between the concentration of plasma viral miRNAs and EBV DNA load in NPC patients [145,148]. While cell-free EBV DNA represents an effective biomarker for NPC diagnosis and prognosis, incorporating miR-BARTs in the analysis may provide additional information to enhance the assessment of the tumor status. We envisage that additional experimental data would warrant a more solid conclusion on this issue.

## Figures and Tables

**Table 1:** Function of EBV miRNAs in viral targets.

miRNA	Target	Function	miRNA effect	References
v-snoRNA^24pp^	BALF5	DNA polymerase	Maintain latency	[76]
BART2-5p	BALF5	DNA polymerase	Maintain latency	[73,102]
BART1-5p/BART16/BART17-5p	LMP1	Transforming factor	Anti-apoptosis	[103]
BART19-5p/BART5	LMP1	Transforming factor	Immune evasion;	[101]
BART9	LMP1	Transforming factor	Promote cell growth (NK/T cells)	[108]
BART22	LMP2A	Signaling molecule	Immune evasion	[23]
BART10-3p	BHRF1	Homolog of Bcl2	Unknown	[101]

**Table 2:** Function of EBV miRNAs in cellular target.

miRNA	Target	Function	miRNA effect	References
BART5-5p	*PUMA*	Proapoptotic protein	Anti-apoptosis	[93]
BARTs cluster 1	*Bim*	Proapoptotic protein	Anti-apoptosis	[110]
BART20-5p	*T-bet* (*TBX21*)	T-box transcription factor	Anti-apoptosis	[111]
BART16	*TOMM22*	Mitochondrial receptor for proapoptotic protein BAX	Anti-apoptosis	[112]
BART1-3p + BART16	*CASP3*	Proapoptotic protein	Anti-apoptosis	[114]
BART13-3p	*CAPRIN2*	Wnt-signaling enhancer	Anti-apoptosis	[101]
BART15	*BRUCE*	Anti-apoptotic protein		[117]
BART15	*NLPR3*	Regulation of inflammation	Immune evasion	[120]
BART2-5p	*MICB*	NK cell ligand	Immune evasion	[125]
BART3	*IPO7*	Nuclear importer protein	Immune evasion	[112]
BART3 + BART16	*IPO7*	Nuclear importer protein	Immune evasion	[114]
BART19-3p	*WIF1*	Wnt inhibitor	Oncogenic properties	[92]
BART19-3p, 7 and 17-5p	*APC*	Wnt inhibitor	Oncogenic properties	[92]
BART19-3p, 14 and 18-5p	*NLK*	Wnt inhibitor	Oncogenic properties	[92]
BART3*	*DICE1*	Tumor suppressor	Oncogenic properties	[128]
BART6-5p	*DICER*	miRNA biogenesis	Unknown	[133]

## Appendix

### Materials and Methods

#### Patient Samples

All specimens in this study were recruited at the Prince of Wales Hospital, Hong Kong. They are included frozen biopsy tissues of 21 NPCs, one EBVaGC and two LELC of lungs, formalin fixed paraffin embedded (FFPE) specimens from 9 cases of EBVaGC, 7 cases of LELC of lung and 7 cases of LELC of CCA. The present of EBV in the samples were confirmed by EBV-encoded small RNA (EBER) *in situ* hybridization (ISH) on the paraffin-embedded tissue sectioned. All participants provided written informed consent for the collection of samples and subsequent analysis. Ethics approval was obtained from the Joint Chinese University of Hong Kong-New Territories East Cluster Clinical Research Ethics Committee, Hong Kong.

#### RNA Extraction and Quantitative Reverse Transcription PCR (QRT-PCR)

Total RNA from biopsies and FFPE samples were prepared by TRIzol reagent (Invitrogen, Carlsbad, CA, USA) and Recover All Total nucleic Acid Isolation kit (Ambion Inc, Austin, TX, USA) according to the manufacturer’s recommendations respectively. The concentration and quality of all RNA was determined by using a NanoDrop-1000 UV-VIS Spectrophotometer (NanoDrop Company, Wilmington, DE, USA). One microgram of total RNA was reverse-transcribed in 20 μL of reaction with miScript Reversed Transcription Kit (Qiagen, Hilden, Germany) and diluted 5-fold for individual QRT-PCR assay. MiScript SYBR green PCR kit (Qiagen, Hilden, Germany) with kit provided universal primer and in-house designed miRNA-specific forward primers were used for QRT-PCR reaction with the following thermal cycling condition: 95 °C for 15 min, followed by 40 cycles at 94 °C for 15 s, annealing with specific temperature for 30 s and 70 °C for 30 s. The PCR was carried out in LightCycler^®^ 480 Real-Time PCR System (Roche Diagnostic) and the crossing point (Cp) was analyzed by second derivative method. To eliminate the non-specific SYBR green signal, PCR result will be normalized with the signal generated by EBV negative epithelial cell line NP69. Standard curves were included in each experiment for absolute quantification of each miRNA per nanogram of total RNA using serially-diluted synthetic DNA-RNA chimeric oligonucleotides (Integrated DNA Technologies, Coralville, IA, USA) with mature miRNA sequences as RNA template (from 10^7^ to 10^3^ copies; 10 μM = 6.02 × 10^12^ copies per micro-liter). It had been demonstrated by other team that standard curve generated from single-stranded oligonucleotides is similar to that obtained by using T7-transcribed RNA [149]. Analysis of each sample was performed in triplicate. The oligonucleotide sequences and specific annealing temperature used for QRT-PCR are shown in Table S1.

#### Expression of BHRF1 and Northern Blot

Expression of BHRF1 mRNA was analyzed by an RT-PCR based assay as previous described [150]. In brief, 1 μg of RNA was reversed transcribed and used to perform PCR reaction with BHRF1 specific primers (5′-GTC AAG GTT TCG TCT GTG TG-3′ and 5′-TTC TCT TGC TGC TAG CTC CA-3′) and actin specific primers (5′-TAA GGA GAA GCT GTG CTA CGT C-3′ and 5′-GGA GTT GAA GGT AGT TTC GTG G-3). PCR products were analyzed in 2% agarose gel and transferred onto Nytran SPC membrane (Whatman Inc, Piscataway, NJ) for Southern blot analysis using γ-32P ATP end-labeled synthetic oligonucleotides complementary to the lytic form of BHRF1 (5′-ATG CAC ACG ACT GTC CCG TAT ACA C-3′). Northern blot was performed as described previously [23,122]. The sequence of synthetic oligonucleotides used as probes for EBER1-5p and 3p were EBER1-5p: 5′-TAG GGC AGC GTA GGT CCT-3′ and EBER1-3p: 5′-AAA CAT GCG GAC CAC CAG CTG G-3′. As a positive control, 5 μg of RNA from EBV-positive marmoset cells line B95-8 was included for Northern blot analysis.

#### AGO2 Co-Immunoprecipitation

The AGO2 co-immunoprecipitation method was described previously [94].

#### Induction of Viral Lytic Cycle in NPC Cell Lines

C666-1 cells were treated with 100 ng/mL of Gemcitabine (GEM, Eli Lilly and Company, IN, USA) and 100 ng/mL Trichostatin A (TSA, Sigma-Aldrich, Saint Louis, MO, USA) for 48 days. The induction of viral lytic cycle in C666-1 cells was confirmed by Western blot analysis using antibodies targeting early viral lytic genes, anti-EA-D (BMRF1, Millipore Corp, Billerica, MA, USA), anti-EBV transcription factor R (BRLF1, Argene, Verniolle, France) and anti-EBV-ZEBRA (Argene, Verniolle, France).

#### Plasmid Constructs

The v-snoRNA1 expression vector were made by inserting the PCR fragments that contains v-snoRNA1 flanking sequence (AJ507799.2, 152761:153032) into pcDNA3.1 expression vector through *Hind III* and *Xba I* sites. The PCR products were amplified using genomic DNA from B95.8 (variant 1), C666-1 (variant 2) and one of the NPC biopsy (variant 3) as template. The sequences of the primes used for the PCR are F: GCC AAG CTT GCC CTT GCG TGT C and R: GGC TCT AGA AGG CTG GCA AAG ATC.

#### *In Vitro* Processing Assay

*In vitro* Drosha/DGCR8 and Dicer digestion assay was described previously [23].

**Table S1:** Oligonucleotide sequences and the specific annealing temperature used for QRT-PCR.

miRNA	Specific forward primer	Chimeric miRNA mimic	Annealing temp.
**BART1-3p**	AGCACCGCTATCCACTATGT	TAGCACCGCTATCCACTATrGrUrC	55
**BART1-5p**	TCTTAGTGGAAGTGACGTGCT	TCTTAGTGGAAGTGACGTGCTrGrUrG	60
**BART2-3p**	AGGAGCGATTTGGAGAAAATAA	AAGGAGCGATTTGGAGAAAATrArArA	60
**BART2-5p**	TATTTTCTGCATTCGCCCTTGC	TATTTTCTGCATTCGCCCTrUrGrC	60
**BART3**	CACCACTAGTCACCAGGTGT	CGCACCACTAGTCACCAGGrUrGrU	60
**BART4**	ACCTGATGCTGCTGGTGTGC	GACCTGATGCTGCTGGTGTrGrCrU	64
**BART5**	AAGGTGAATATAGCTGCCCAT	CAAGGTGAATATAGCTGCCCArUrCrG	55
**BART6-3p**	GGGATCGGACTAGCCTTAGA	CGGGGATCGGACTAGCCTTrArGrA	55
**BART6-5p**	TAAGGTTGGTCCAATCCATAGG	TAAGGTTGGTCCAATCCATrArGrG	55
**BART7**	CATCATAGTCCAGTGTCCAGGG	CATCATAGTCCAGTGTCCArGrGrG	60
**BART8**	TACGGTTTCCTAGATTGTACAG	TACGGTTTCCTAGATTGTArCrArG	55
**BART9**	TAACACTTCATGGGTCCCGTAGT	TAACACTTCATGGGTCCCGTrArGrU	55
**BART10**	TACATAACCATGGAGTTGGCTGT	TACATAACCATGGAGTTGGCrUrGrU	60
**BART11-3p**	ACGCACACCAGGCTGACTG	ACGCACACCAGGCTGACTrGrCrC	62
**BART11-5p**	AGACAGTTTGGTGCGCTAGT	TCAGACAGTTTGGTGCGCTAGrUrUrG	55
**BART12**	CTGTGGTGTTTGGTGTGGTT	TCCTGTGGTGTTTGGTGTGrGrUrU	62
**BART13**	ACACTCCAGCTGGGTGTAACTTGCCAGGGA	TGTAACTTGCCAGGGACGGCrUrGrA	62
**BART14**	TAAATGCTGCAGTAGTAGGGA	TAAATGCTGCAGTAGTAGGrGrArU	55
**BART15**	TCAGTGGTTTTGTTTCCTTGA	GTCAGTGGTTTTGTTTCCTrUrGrA	60
**BART16**	TAGATAGAGTGGGTGTGTGCTC	TTAGATAGAGTGGGTGTGTGCrUrCrU	64
**BART17-3p**	GTATGCCTGGTGTTCCCCTTA	TGTATGCCTGGTGTCCCCTTrArGrU	60
**BART17-5p**	TAAGAGGACGCAGGCATACA	TAAGAGGACGCAGCATACrArArG	55
**BART18-3p**	TATCGGAAGTTTGGGCTTCGT	TATCGGAAGTTTGGGCTTCrGrUrC	60
**BART18-5p**	TCAAGTTCGCACTTCCTATAC	TCAAGTTCGCACTTCCTATrArCrA	55
**BART19-3p**	ACACTCCAGCTGGGUUUUGUUUGCUUGGGA	TTTTGTTTGCTTGGGAATrGrCrU	55
**BART19-5p**	ACATTCCCCGCAAACATGACAT	ACATTCCCCGCAAACATGACrArUrG	55
**BART20-3p**	CATGAAGGCACAGCCTGTTAC	CATGAAGGCACAGCCTGTTrArCrC	60
**BART20-5p**	TAGCAGGCATGTCTTCATTCC	TAGCAGGCATGTCTTCATrUrCrC	60
**BART21-5p**	CACTAGTGAAGGCAACTAAC	TCACTAGTGAAGGCAACTrArArC	55
**BART22**	TTACAAAGTCATGGTCTAGTAGT	TTACAAAGTCATGGTCTAGTrArGrU	55

Figure S1 Lack of association between novel EBER end terminal fragments and AGO2: (**A**) Expression of small EBER fragments in NPCs was validated by Northern blot analysis. Locations of the EBER1-5p and −3p fragments are indicated with arrow; (**B**) Western blot analysis of hAGO2 protein from C666-1 whole cell lysate before (Input) and after immunoprecipitation (IP) with anti-AGO2 antibody (*left panel*). Monoclonal antibody against FLAG tag (lane 2) and against hAGO2 4 (lane 3) were used for IP from C666-1 cell lysate (lane 1). AGO-2 associated miRNAs were analyzed by Northern blot analysis with synthetic oligonucleotide probe for the specific small EBV fragments listed below the figure. Locations of the probed fragments are indicated with arrows. RNA loading was visualized by SYBR Gold stained PAGE. BART22 IP was included as positive control. The information of EBER1-5p and 3p were described in a previous publication [23].

Figure S2 Absence of miR-BHRF1s expression in epithelial cancers: (**A**) Expression of BHRF1 mRNA transcripts in NPC cell lines (lanes 2–6), B-cell cell lines (lanes 7–10), NPC xenografts (lanes 11–12) and NPC biopsies (lanes 13–16) was analyzed by RT-PCR analysis. The RT-PCR products were separated on agarose gel (*left upper panel*) and transferred to a membrane for detection by Southern blot (*left middle panel*). Expression of actin in RT-PCR was used as a control (*left lower panel*). Representative Northern blot results showed the expression of ebv-miRNAs in different EBV positive samples (*right panel*); (**B**) Induction of EBV lytic cycles in C666-1 cells was confirmed by Western blot analysis (*left panel*). Expression of miR-BHRF1 and hsa-miR-16 was demonstrated by Northern blot analysis (*right panel*); (**C**) Representative Northern blot analysis on RNA samples from different EBV positive epithelial carcinomas was performed. The biopsies included NPC, Lymophoepithelial-like cholangiocarcinoma (LEL-CCA) and Lymphoepithelial-like carcinoma of the lung (LELC-Lung).

Figure S3 The predicted secondary structures of the long v-snoRNA1 containing fragment. The RNA folding of EBV fragment (AJ507799.2, 152761:153032) predicted by MFOLD are shown in B95.8-EBV (Variant 1) (*left panel*); C666-1-EBV (Variant 2) (*middle panel*) and the common EBV strain in our locality (Variant 3) (*right panel*). The folding energy (dG) with units (kcal/mol) is indicated. The location of v-snoRNA1 is indicated by sequences between red arrows.
