# Supplementary figures and images for: Emerging Roles of Small Epstein-Barr Virus Derived Non-Coding RNAs in Epithelial Malignancy
Source: Int J Mol Sci. 2013 Aug 23;14(9):17378–409. doi: 10.3390/ijms140917378 (PMC3794732; doi:10.3390/ijms140917378)

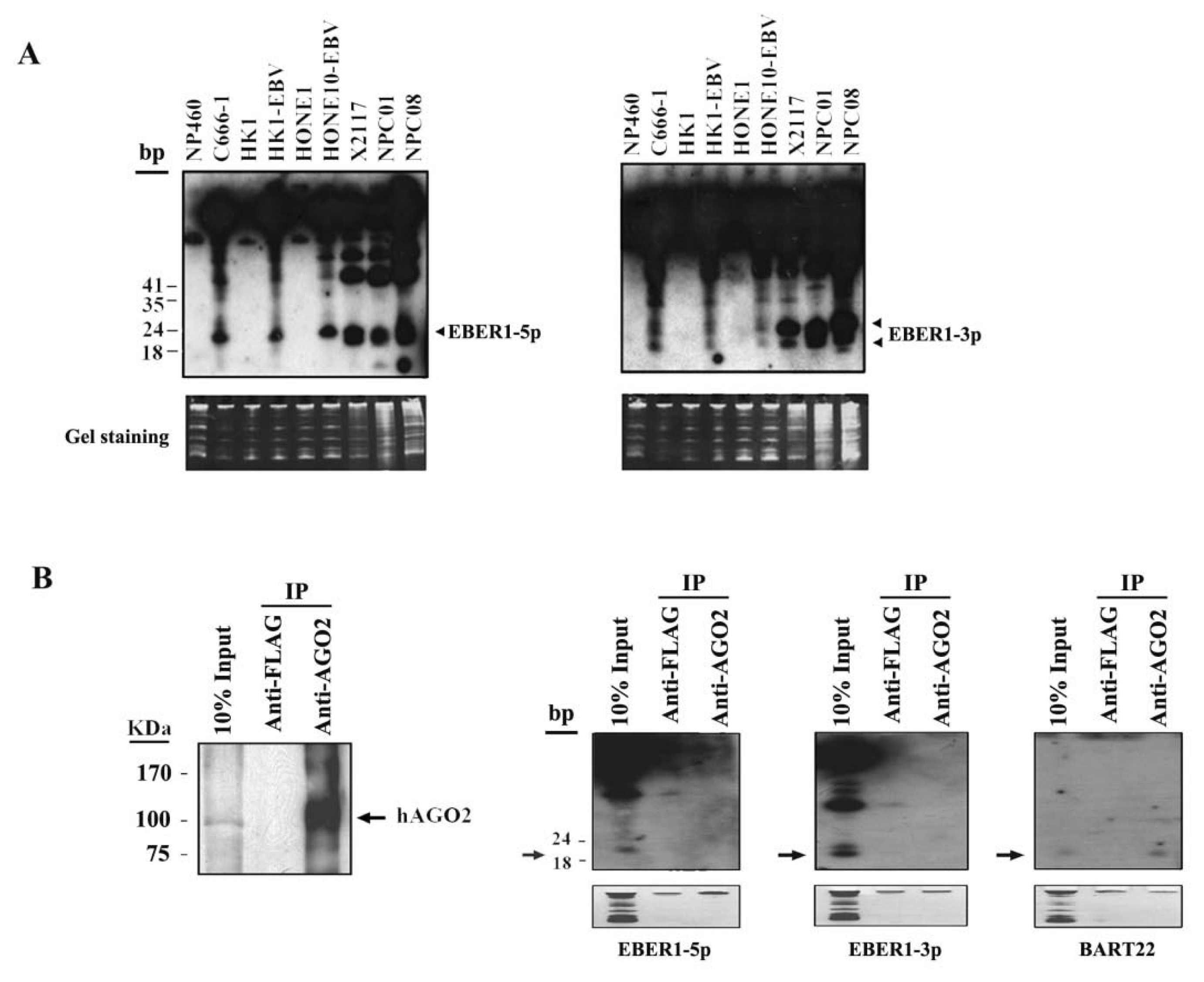

Supplement: Figure S1 — Lack of association between novel EBER end terminal fragments and AGO2: (A) Expression of small EBER fragments in NPCs was validated by Northern blot analysis. Locations of the EBER1-5p and −3p fragments are indicated with arrow; (B) Western blot analysis of hAGO2 protein from C666-1 whole cell lysate before (Input) and after immunoprecipitation (IP) with anti-AGO2 antibody (left panel). Monoclonal antibody against FLAG tag (lane 2) and against hAGO2 4 (lane 3) were used for IP from C666-1 cell lysate (lane 1). AGO-2 associated miRNAs were analyzed by Northern blot analysis with synthetic oligonucleotide probe for the specific small EBV fragments listed below the figure. Locations of the probed fragments are indicated with arrows. RNA loading was visualized by SYBR Gold stained PAGE. BART22 IP was included as positive control. The information of EBER1-5p and 3p were described in a previous publication [23]. [file ijms-14-17378s1.tif]

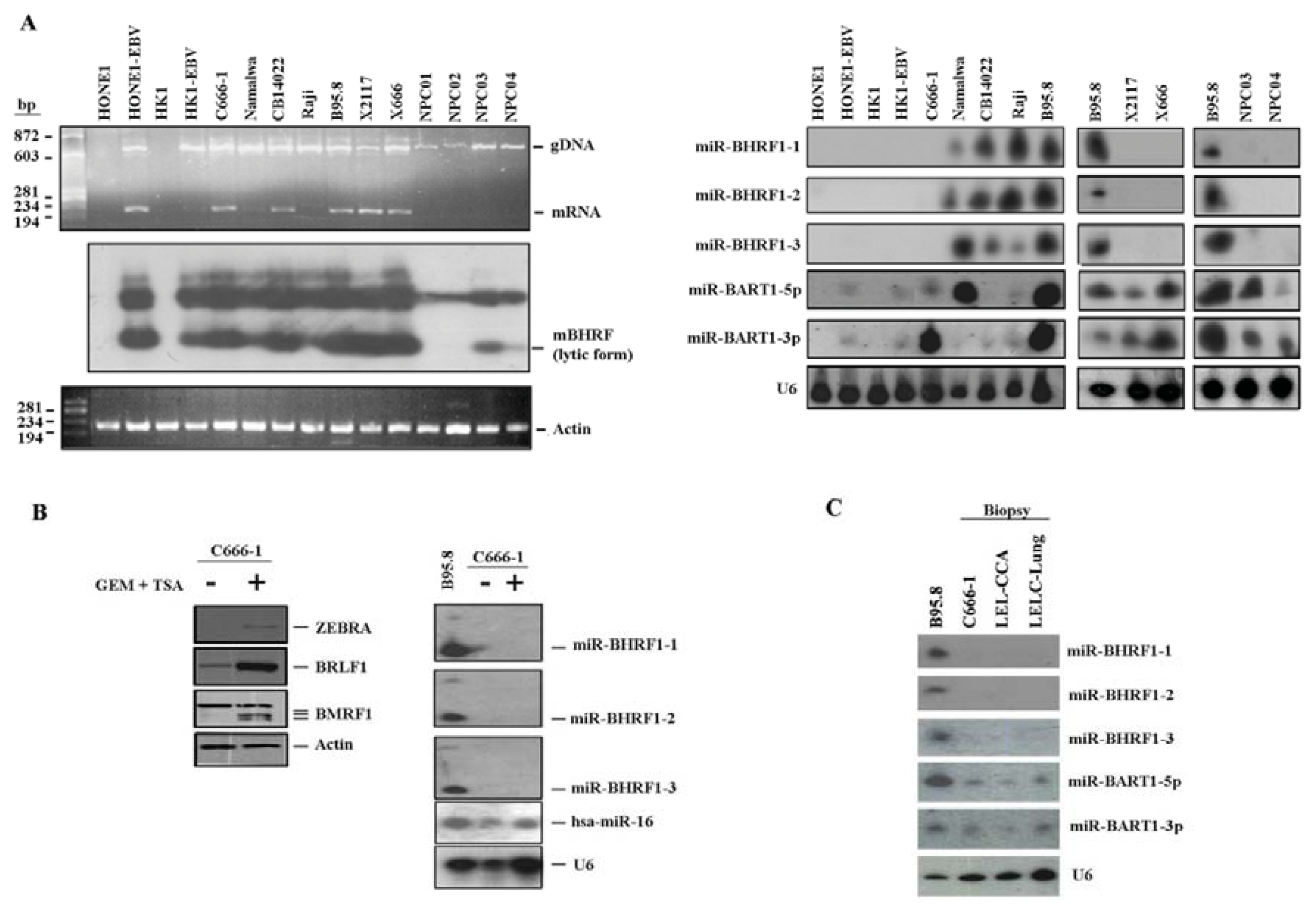

Supplement: Figure S2 — Absence of miR-BHRF1s expression in epithelial cancers: (A) Expression of BHRF1 mRNA transcripts in NPC cell lines (lanes 2–6), B-cell cell lines (lanes 7–10), NPC xenografts (lanes 11–12) and NPC biopsies (lanes 13–16) was analyzed by RT-PCR analysis. The RT-PCR products were separated on agarose gel (left upper panel) and transferred to a membrane for detection by Southern blot (left middle panel). Expression of actin in RT-PCR was used as a control (left lower panel). Representative Northern blot results showed the expression of ebv-miRNAs in different EBV positive samples (right panel); (B) Induction of EBV lytic cycles in C666-1 cells was confirmed by Western blot analysis (left panel). Expression of miR-BHRF1 and hsa-miR-16 was demonstrated by Northern blot analysis (right panel); (C) Representative Northern blot analysis on RNA samples from different EBV positive epithelial carcinomas was performed. The biopsies included NPC, Lymophoepithelial-like cholangiocarcinoma (LEL-CCA) and Lymphoepithelial-like carcinoma of the lung (LELC-Lung). [file ijms-14-17378s2.tif]

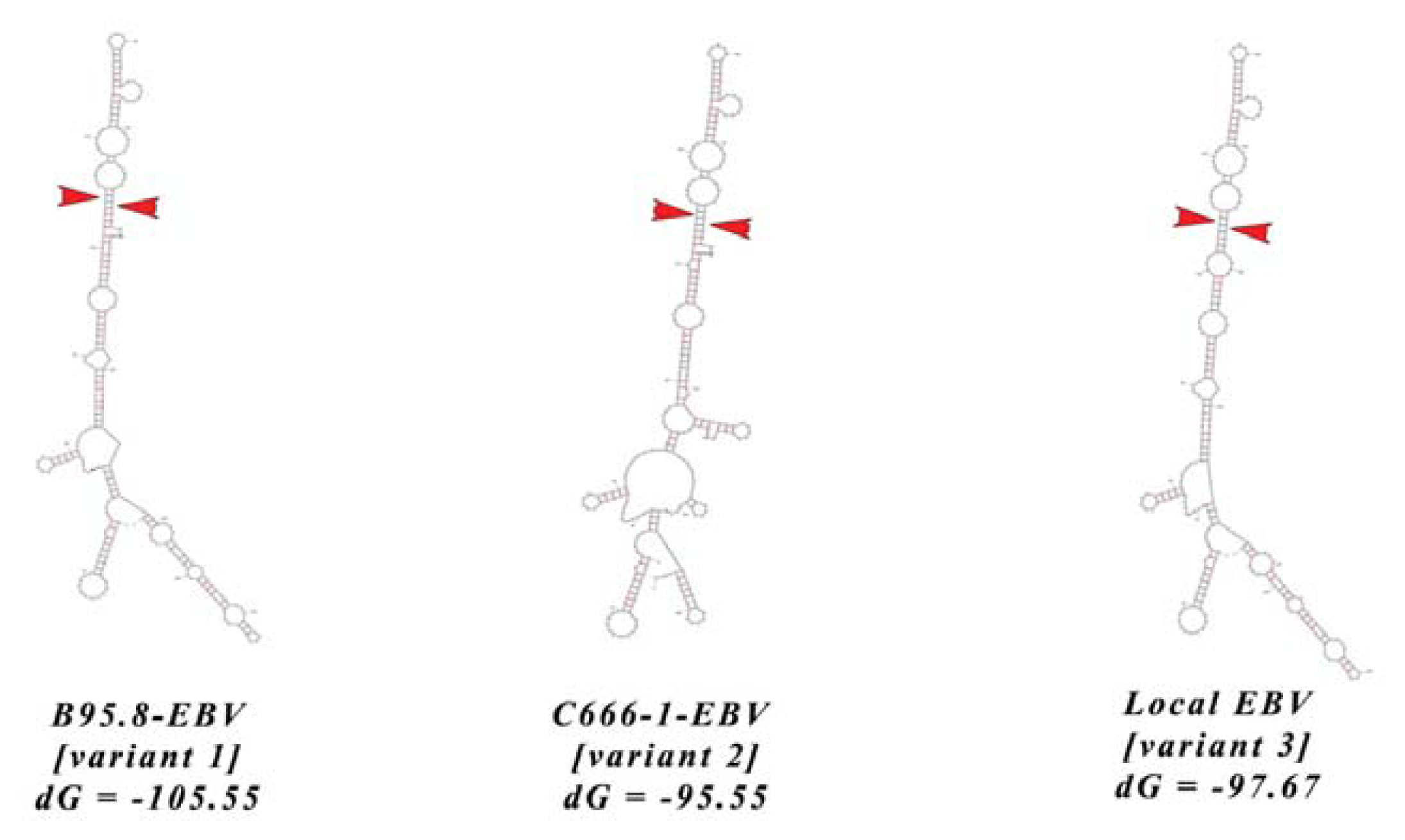

Supplement: Figure S3 — The predicted secondary structures of the long v-snoRNA1 containing fragment. The RNA folding of EBV fragment (AJ507799.2, 152761:153032) predicted by MFOLD are shown in B95.8-EBV (Variant 1) (left panel); C666-1-EBV (Variant 2) (middle panel) and the common EBV strain in our locality (Variant 3) (right panel). The folding energy (dG) with units (kcal/mol) is indicated. The location of v-snoRNA1 is indicated by sequences between red arrows. [file ijms-14-17378s3.tif]
